# Laboratory Diagnosis of Respiratory Tract Infections in Children – the State of the Art

**DOI:** 10.3389/fmicb.2018.02478

**Published:** 2018-10-18

**Authors:** Shubhagata Das, Sherry Dunbar, Yi-Wei Tang

**Affiliations:** ^1^Global Scientific Affairs, Luminex Corporation, Austin, TX, United States; ^2^Department of Laboratory Medicine, Memorial Sloan Kettering Cancer Center, Weill Medical College of Cornell University, New York, NY, United States; ^3^Department of Pathology and Laboratory Medicine, Memorial Sloan Kettering Cancer Center, Weill Medical College of Cornell University, New York, NY, United States

**Keywords:** respiratory tract infection, respiratory virus, pediatric population, respiratory infection diagnosis, molecular diagnostics

## Abstract

In the pediatric population, respiratory infections are the most common cause of physician visits. Although many respiratory illnesses are self-limiting viral infections that resolve with time and supportive care, it can be critical to identify the causative pathogen at an early stage of the disease in order to implement effective antimicrobial therapy and infection control. Over the last few years, diagnostics for respiratory infections have evolved substantially, with the development of novel assays and the availability of updated tests for newer strains of pathogens. Newer laboratory methods are rapid, highly sensitive and specific, and are gradually replacing the conventional gold standards, although the clinical utility of these assays is still under evaluation. This article reviews the current laboratory methods available for testing for respiratory pathogens and discusses the advantages and disadvantages of each approach.

## Introduction

Acute respiratory tract infections are one of the leading causes of childhood morbidity and mortality worldwide, and it has been estimated that globally, respiratory infections are responsible for about 2 million deaths in children between 0 and 5 years of age (Bryce et al., 2005; Kallander et al., 2008). Approximately 80% of these respiratory infection cases are caused by viral pathogens such as influenza A and B, respiratory syncytial virus (RSV) A and B, parainfluenza virus types 1–3, adenovirus, rhinovirus, human metapneumovirus (hMPV), and others (Mahony, 2008). The non-specific clinical presentation of respiratory infections poses a considerable challenge to the differential diagnosis of these pathogens. Early and accurate diagnosis of the causative pathogens in respiratory infections is essential to administer appropriate antiviral or antibacterial therapy, initiate effective infection control measures, and reduce the length of hospital stay (Barenfanger et al., 2000; Byington et al., 2002; Akers et al., 2017). In the last decade, there has been a remarkable improvement in the diagnosis of respiratory pathogens with the availability of molecular and point-of-care (POC) testing. Although an increasing number of laboratories are adopting rapid molecular assays, conventional testing methods, such as culture and immunodiagnostics are still used. This review focuses on current laboratory methods for testing for respiratory pathogens, and discusses the advantages and disadvantages of each approach.

## Non-Molecular Methods for Detection and Identification of Respiratory Pathogens

### Electron Microscopy

Electron microscopy (EM) is one of the oldest direct examination methods that has been implemented for both clinical viral diagnosis and study of viral ultrastructure and pathogenesis in developed countries (Roingeard, 2008). Historically, EM has played an instrumental role in identifying novel viral strains in several outbreak situations, such as the coronavirus associated with the severe acute respiratory syndrome (SARS) outbreak (Falsey and Walsh, 2003; Ksiazek et al., 2003). However, despite several advantages, the use of EM has been limited in respiratory viral diagnosis as it is expensive, laborious, time-consuming, and has a greater turnaround time (approximately 3–16 h including specimen preparation), and is often insensitive when compared to other diagnostic methods (Goldsmith and Miller, 2009; Zhang et al., 2013). Additionally, EM requires strict control of experimental conditions, a high concentration of viral particles (> 10^5^ L^-1^), and considerable technical skill and expertise for accurate analysis.

### Culture

Detection of viruses by observing the cytopathic effect and hemadsorption in cell culture has been considered the “gold standard” for diagnosis of respiratory viral pathogens for decades. Viruses such as adenovirus, influenza A/B, RSV, and human parainfluenza viruses are the most common respiratory viruses that are isolated and detected by cell culture (Olsen et al., 1993; Winn and Koneman, 2006). The traditional tube culture method is advantageous for growing a wide variety of viruses, including novel or unknown viruses, but takes days and often weeks to provide results. Over the years, modified cell culture methods such as the centrifugation enhanced shell-vial method has reduced the turnaround time from 5 to 10 days to 24 h (Dilnessa and Zeleke, 2017). Shell-vial culture using combination cell lines allows simultaneous detection of multiple respiratory viruses and, as compared to conventional culture, has similar sensitivity for parainfluenza 1-3 (87% vs. 83%) and influenza A/B (78% vs. 75%), and significantly higher sensitivity for RSV (73% vs. 42%) (LaSala et al., 2007). Despite these advantages, many clinically relevant viruses are difficult to grow in culture (such as rhinovirus and coronavirus) and may produce variable results (Ieven and Goossens, 1997; Hodinka, 2013). Additionally, multiple freezing and thawing of the samples prior to testing can reduce the viral titer, thus effecting the growth in culture. Therefore, as compared to molecular tests, both traditional tube and shell-vial culture methods are laborious, exhibit higher false negative rates, and have longer turnaround times, making viral culture less clinically relevant (Leland and Ginocchio, 2007; Ginocchio, 2007; Hematian et al., 2016).

Culture is also the gold standard for detection of atypical (bacterial) respiratory pathogens, which is followed by identification and antimicrobial susceptibility testing by various manual or automated methods (Tenover, 2011). Bacterial culture can also often be insensitive, especially when specimen adequacy is not determined by Gram stain or if specimens were collected after antibiotic exposure (ATS and IDSA, 2005; Woodhead et al., 2005; Harris et al., 2017). Bacterial culture is also labor intensive, requires substantial technical expertise, and has a typical turnaround time of 48 – 96 h if antibiotic susceptibility testing is performed, and can therefore be considered inadequate for optimal patient care and guiding effective antimicrobial therapy (Tenover, 2011).

### Antigen Detection Assays

#### Rapid Immunoassays

Rapid immunoassays (RIAs) can deliver test results in less than 30 min and are usually performed in the POC setting, thus allowing the test results to be incorporated into the clinical decision-making algorithm (Weinberg and Walker, 2005). Among the four primary RIA formats (latex agglutination, horizontal flow devices, lateral flow devices, and optical immunoassays), the lateral-flow immunoassay (LFIA) is the most versatile and popular immunochromatographic method. RIAs are relatively inexpensive, easy to perform, and most of them have waived status in the United States according to the Clinical Laboratory Improvement Act (CLIA) guidelines, thereby making them invaluable in outpatient clinics, primary care, emergency, and low resource settings (Ginocchio, 2007). Currently, commercially available RIAs are mostly limited to the detection of influenza A virus, influenza B virus, and RSV. Numerous studies have revealed that RIAs demonstrate overall poor sensitivity for influenza and RSV (44–95%); however, they have a higher median specificity (90 to 95%) as compared to cell culture (WHO, 2005; Leland and Ginocchio, 2007; Ginocchio, 2007).

In the pediatric population, commercially available immunoassays have demonstrated high sensitivity (93%) for detection of RSV, and a systematic review of published studies has further revealed that the sensitivity of RSV RIAs is relatively higher for children (81%) than adults (29%) (Slinger et al., 2004; Chartrand et al., 2015). The higher sensitivity can be attributed to the fact that pediatric patients often shed higher titers of respiratory viruses and for a longer time as compared to adults (Englund et al., 1996; Kawai et al., 2007). Despite the overall lower sensitivity, RIAs have been deemed a valuable diagnostic tool in the emergency department as they can significantly decrease the length of stay, additional ancillary testing, and antibiotic prescriptions for those children who do test positive for influenza (Abanses et al., 2006; Blaschke et al., 2014).

#### Direct Fluorescent Antibody Tests

Direct fluorescent antibody (DFA) testing of nasopharyngeal wash specimens is considered a rapid and reliable method for detecting respiratory viral infections. Commercial DFA kits have demonstrated high sensitivity and specificity for multiple respiratory viruses such as hMPV (95 and 100%), adenovirus (62 and 100%), RSV (94 and 96%), and parainfluenza viruses (88 and 99.7%), although the results can be subjective and require technical expertise for accurate interpretation (Landry and Ferguson, 2000; Ohm-Smith et al., 2004; Rocholl et al., 2004; Aslanzadeh et al., 2008; Vinh et al., 2008). The high specificity (99–100%) of DFA indicates that the test can be used as a reliable detection method, especially during the initial days of the illness, as was shown by Shafik et al. (2011) for RSV in children.

### Serological Tests

Pathogen-specific antibodies typically appear about 2 weeks after the initial infection and can be detected by serological tests. Serological tests can successfully identify antibodies to most respiratory pathogens such as RSV, adenovirus, influenza A and B, parainfluenza 1-3 virus, etc., and can detect mixed infections from hospitalized children suffering from acute respiratory infections, with the exception of infants for whom an antibody response is usually undetected (Hall et al., 1991; Chkhaidze et al., 2006). However, it has been reported that serological assays are significantly less sensitive for the detection of parainfluenza virus and adenovirus when compared to molecular methods, such as RT-PCR (Kuypers et al., 2006). Kuypers et al. (2006) found that RT-PCR detected 40% more specimens from pediatric patients that were positive for at least one respiratory virus than were detected by fluorescent antibody assay (FA). FA testing, in addition to RT-PCR, is useful for epidemiological studies as it increases the probability of identifying acute viral infections and has been used for accurate assessment of respiratory viruses other than influenza in children (Sawatwong et al., 2012; Feikin et al., 2013; Zhang et al., 2017).

For bacteria, serological testing is particularly challenging, especially for identifying atypical bacterial agents such as *Mycoplasma pneumoniae*. With varied sensitivity (14% – 77%) and specificity (49% – 97%) as compared to PCR, serological testing has limited usefulness clinically (Beersma et al., 2005; Wellinghausen et al., 2006). The clinical utility of serologic tests is further limited because they require both acute and convalescent sera to monitor seroconversion or to identify a four-fold increase in antibody titer (Loeffelholz and Chonmaitree, 2010). Additionally, serological tests are not relevant for identifying frequently recurring viral infections as the serum IgM levels are lower due to repeated exposure to vaccines or circulating viruses. For optimal virus specific IgM testing, an acute-phase serum specimen should be obtained early in the course of illness (Dunn, 2015).

## Nucleic Acid Amplification Tests for Detection and Identification of Respiratory Pathogens

A wide variety of newer, nucleic acid amplification tests (NAATs) for the detection of respiratory pathogens are commercially available. These tests are listed in **Table 1** and described below according to complexity and pathogen coverage. The accuracy of respiratory virus detection by molecular tests is not only dependent on their specific assay chemistry, but is also critically affected by the type, quantity, and quality of specimens collected (Dunn, 2015). Several types of specimens can be used for detection of respiratory viruses, including: bronchoalveolar lavage (BAL), throat swab, nasopharyngeal (NP) washes, NP aspirates, lung aspirates, and NP swabs, although the appropriate specimen type depends on the specific patient population. For example, in pediatric patients, obtaining nasal washes and nasal aspirates is more technically challenging when compared to NP swabs, especially in severely ill children and in poor resource settings (Hammitt et al., 2012). It has been observed that for optimal test results, specimens should be collected within 3–5 days after onset of symptoms to ensure that the sample has a high concentration of virus particles, viral antigen or viral nucleic acids, should be transported to the testing laboratory in refrigerated condition (2–8°C) in appropriate transport media, and testing should be performed within 48 h (Grys and Smith, 2009).

**Table 1:** Commercially available nucleic acid amplification assays for respiratory pathogens^a^.

Test	Manufacturer	Technology	Targets	Sample type^b^	Turnaround time	Complexity classification
NxTAG^®^ respiratory pathogen panel	Luminex Corporation	Real-time RT-PCR	Multiplex Panel (20 targets)	NPS	∼4 h	High
eSensor^®^ respiratory viral panel (RVP)	GenMark Diagnostics	Multiplex microarray, competitive DNA hybridization	Multiplex Panel (14 targets)	NPS	∼8 h	High
Verigene^®^ RP flex	Luminex Corporation	RT-PCR & and microarray hybridization	Multiplex Panel (16 targets)	NPS	∼2 h	Moderate
ePlex^®^ respiratory pathogen (RP) panel	GenMark Diagnostics	RT-PCR	Multiplex Panel (17 targets)	NPS	∼2 h	Moderate
FilmArray^®^ respiratory panel (RP)	BioFire Diagnostics, Inc.,	Nested multiplex RT-PCR	Multiplex Panel (20 targets)	NPS	∼1 h	Moderate
FilmArray^®^ respiratory panel 2 (RP2)	BioFire Diagnostics, Inc.,	Nested multiplex RT-PCR	Multiplex Panel (21 targets)	NPS	∼45 min	Moderate
FilmArray^®^ respiratory panel^®^ (RP) EZ	BioFire Diagnostics, Inc.,	Nested multiplex RT-PCR	Multiplex Panel (14 targets)	NPS	∼1 h	Waived
Lyra^®^ parainfluenza virus assay	Quidel Corporation	Real-time RT-PCR	Parainfluenza virus types 1, 2, and 3	NS, NPS	∼4 h	High
Lyra^®^ RSV + hMPV assay	Quidel Corporation	Real-time RT-PCR	RSV, hMPV	NS, NPS	∼4 h	High
Simplexa^TM^ flu A/B & RSV Kit	Focus Diagnostics, Inc.,	Real-time RT-PCR	Flu A, Flu B, and RSV	TS	<4 h	High
Panther fusion flu A/B/RSV	Hologic, Inc.,	Real-time RT-PCR	Flu A, Flu B, and RSV	NPS	∼2.5 h	High
Panther fusion paraflu assay	Hologic, Inc.,	Real-time RT-PCR	Parainfluenza 1, 2, and 3	NPS	∼2.5 h	High
Panther fusion AdV/hMPV/RV assay	Hologic, Inc.,	Real-time RT-PCR	Adenovirus, hMPV, and Rhinovirus	NPS	∼2.5 h	High
ARIES^®^ flu A/B & RSV assay	Luminex Corporation	Real-time PCR	Flu A, Flu B, and RSV	NPS	<2 h	Moderate
ARIES^®^ bordetella assay	Luminex Corporation	Real-time PCR	*B. pertussis, B. parapertussis*	NPS	<2 h	Moderate
Simplexa^TM^ flu A/B & RSV direct	Focus Diagnostics, Inc.,	Real-time RT-PCR	Flu A, Flu B, and RSV	NPS	<2 h	Moderate
Xpert^®^ flu/RSV XC	Cepheid	Real-time RT-PCR	Flu A, Flu B, and RSV	NPS, NA, and NW	<1 h	Moderate
Solana RSV + hMPV assay	Quidel Corporation	Isothermal RT-helicase-dependent amplification (HDA)	RSV, hMPV	NS, NPS	∼45 min	Moderate
Illumigene^®^ mycoplasma direct DNA amplification assay	Meridian Bioscience, Inc.,	Loop-mediated isothermal DNA amplification (LAMP)	*Mycoplasma pneumoniae*	NPS, TS	<1 h	Moderate
Illumigene pertussis DNA amplification assay	Meridian Bioscience, Inc.,	Loop-mediated isothermal DNA amplification (LAMP)	*Bordetella pertussis*	NPS	<1 h	Moderate
Xpert^®^ xpress Flu/RSV	Cepheid	Real-time RT-PCR	Flu A, Flu B, and RSV	NPS, NA, and NW	∼30 min	Waived
Xpert^®^ xpress flu	Cepheid	Real-time RT-PCR	Flu A, Flu B	NPS, NA, and NW	∼30 min	Waived
cobas^®^ Liat influenza A/B & RSV assay	Roche Molecular Diagnostics	Real-time RT-PCR	Flu A, Flu B, and RSV	NPS	∼20 min	Waived
cobas^®^ Liat influenza A/B assay	Roche Molecular Diagnostics	Real-time RT-PCR	Flu A, Flu B	NPS	∼20 min	Waived
Alere i influenza A & B 2 test	Abbott Laboratories	Isothermal nucleic acid amplification	Flu A, Flu B	NPS, NS	<15 min	Waived
Alere i RSV	Abbott Laboratories	Isothermal nucleic acid amplification	RSV	NPS, NPS in VTM	<15 min	Waived


*^*a*^FDA 510(k) Premarket Notification (FDA, 2018a). Retrieved from https://www.accessdata.fda.gov/scripts/cdrh/cfdocs/cfpmn/pmn.cfm; ^*b*^NPS, nasopharyngeal swab; NS, nasal swab; NA, nasal aspirate; NW, nasal wash; TS, throat swab. ^*c*^Waived for direct NS, NPS, and NA/NW specimens; Moderate for NS and NA/NW specimens eluted in transport media.*

### High Complexity Multiplex Panel Assays

Detection of respiratory pathogens by NAATs such as PCR, nucleic acid sequence-based amplification (NASBA), transcription mediated amplification (TMA), strand displacement amplification (SDA), loop-mediated isothermal amplification (LAMP), rolling circle amplification (RCA), etc., have gained immense popularity over the past decade. Large syndromic panels that can detect multiple pathogens simultaneously are beneficial for infection control, timely treatment decisions, and are substantially less expensive than detecting individual pathogens by monoplex real-time RT-PCR (Reijans et al., 2008; Schreckenberger and McAdam, 2015). These multiplex respiratory panels vary in terms of their complexity (high, moderate, or waived), throughput, pathogens detected, ability to subtype, instrumentation (batched or random access), workflow (stepwise or integrated sample-to-answer), ease of use, and time to result (several hours to minutes) (Caliendo, 2011; Krunic et al., 2011; Mahony et al., 2011). Studies have reported increased diagnostic yield (60% vs. 35%) and considerably higher sensitivity (80–100%) and specificity (82–100%) for these assays when compared to conventional diagnostic methods such as DFA, viral isolation, and immunoassays (Reijans et al., 2008; Gharabaghi et al., 2011).

In comparison to other NAAT-based molecular methods, high-plex syndromic panels demonstrate comparable performance, although sensitivity and specificity might vary for individual targets (Sails et al., 2017). For example, greater than 99% specificity was observed for all targets except enterovirus/rhinovirus (94%), and the overall sensitivity ranged between (83–100%) when the NxTAG RPP and the Anyplex II RV16 assays were evaluated for detection of respiratory pathogens in hospitalized children (Brotons et al., 2016). Syndromic panels can also detect bacterial infections and viral-bacterial coinfections, which is vital for developing effective treatment plans and prevention strategies (Brealey et al., 2015; Brotons et al., 2016). However, for novel pathogen strains, such as Flu A(H1N1pdm2009), panel tests often fail to identify the subtype and require confirmation by other tests (Bryce et al., 2012).

Syndromic respiratory panels with high sample throughput can usually run 1–96 specimens and are mostly classified as high complexity assays according to CLIA guidelines, which indicates that running these panel requires considerable knowledge, training, and experience (FDA, 2018b). It has been noted that these high throughput panels are ideal for batch testing, especially during outbreak situations and respiratory viral seasons when sample volumes are unusually high (Ginocchio et al., 2009; Crawford et al., 2010; Tang et al., 2016). Despite the advantages, implementation of high throughput panels can be challenging because of demanding sample preparation, processing, and result interpretation procedures, and the turnaround time varies from approximately 5–16 h (Beckmann and Hirsch, 2016).

### Moderate Complexity Multiplex Integrated Systems

Moderate complexity sample-to-answer molecular test systems have gained immense popularity over the last few years because of their ease of use, faster turnaround time, and efficient workflow. Currently, the United States. FDA-cleared sample-to-answer test systems include both low-plex, targeted assays and high-plex panels that can detect multiple pathogens and pathogen classes. These tests usually have low to moderate sample throughput (e.g., 1–12 samples/run), but have faster turnaround times when compared to batched, high throughput, multiplex panels (Popowitch et al., 2013). The high-plex, sample-to-answer panels can detect up to 12–20 pathogens based on the panel size. Most of these qualitative assays allow random access that ensures that tests can be performed on demand, and the reactions occur in closed cassettes or cartridges, thereby minimizing the risk of contamination (Poritz et al., 2011; Popowitch and Miller, 2015).

Comparative studies evaluating the performance of these assays has reported more than 95% agreement between different commercial platforms, and greater than 85% sensitivity and 99% specificity in most cases (Babady et al., 2018). However, some high-plex sample-to-answer tests have demonstrated lower sensitivity for certain pathogens such as adenovirus (57%), influenza A(H1N1pdm09) (73%), and influenza B virus (77%) (Popowitch et al., 2013). Cross-reactivity between adenovirus subtypes has also been reported for some assays, but the differentiation of these groups is not deemed clinically important for routine diagnosis (Lin et al., 2004; Gray et al., 2007).

Despite a short turnaround time of 1–2 h, the clinical utility of these assays has been questioned since no statistically significant reduction in antibiotic prescription rates or mortality has been observed between patients who tested positive for a non-influenza virus and those who tested negative (48% vs. 49% respectively) (Green et al., 2016). In some cases, a reduction in length of hospital stay (7 days vs. 9 days) and duration of isolation (75 h vs. 82 h) was observed (Rogers et al., 2015; Akers et al., 2017). The primary concerns for the high-plex sample-to-answer assays are high cost per test, and the inability to order customized, targeted panels (Ramanan et al., 2018). Multiplex assays such as the Verigene RP *Flex* panel (Luminex) offer flexible testing and payment options by allowing users to order any combination of targets for an individual sample.

### Low-Plex Integrated Test Systems

Low-plex integrated respiratory test systems typically target 1–4 pathogens per assay. Comparative studies evaluating the performance of these assays has reported more than 90% concordance between different commercial platforms, and greater than 95% sensitivity and specificity in most cases (Dugas et al., 2014; Selvaraju et al., 2014; McMullen et al., 2017). The total turnaround time per sample for these assays varies between ∼1–2 h, and the number of samples that can be tested in a standard 8-h work shift depends on the number of instruments available in the testing laboratory (Ling et al., 2018). Smaller panels targeting a few pathogens that can be run independently (random access) or simultaneously (random batch) could be a viable option for controlling laboratory costs. Therefore, when considering among various testing options, it is recommended that in addition to clinical performance, other assay characteristics such as ease of use, panel composition, total turnaround time, hands-on time, and cost be considered before implementing an assay in various clinical settings (Juretschko et al., 2017).

### Waived Molecular Point-of-Care Tests

Nucleic acid amplification based POC testing is relatively new in the realm of respiratory viral diagnosis and has generated contradicting opinions regarding their implementation and clinical utility. Molecular POC assays have extremely short turnaround times (<30 min), minimal hands-on time (1–2 min), and can be easily operated by non-laboratory staff members, thereby making them suitable for near patient implementation and testing (Brendish et al., 2015; Peters et al., 2017; Ling et al., 2018). Currently most of the United States. FDA-cleared commercial POC assays are limited to the detection of influenza A/B and RSV viruses, with the exception of the FilmArray^®^ RP EZ (BioFire) which detects 14 targets (FDA, 2018c). Most of the studies evaluating the clinical performance of these assays have reported high sensitivity (87–100%) and specificity (>98%) for detecting influenza A/B and RSV in pediatric and adult patients (Bell et al., 2014; Nie et al., 2014; Popowitch and Miller, 2015; Gibson et al., 2017; Ling et al., 2018). However, these studies have also shown that the clinical performance varies and sensitivity is significantly low for influenza B (45.2–54.5%); therefore, the results should be interpreted with caution and additional confirmatory testing should be considered, depending on the clinical circumstances (Jokela et al., 2015; Busson et al., 2017; Davis et al., 2017). Molecular POC tests can be costly and more prone to incorrect results and contamination, as the assays are usually performed by non-laboratory personnel and poor training, failure to follow manufacturer’s instructions, and failure to perform adequate quality control might affect the accuracy of results (Azar and Landry, 2018). Currently, more evidence-based studies are required to understand the overall clinical utility of molecular POC testing, since no correlation has been observed between implementation of these tests and reduction of antibiotic usage or hospital length of stay (Andrews et al., 2017; Brendish et al., 2017).

## Concluding Remarks

Molecular testing has considerably improved the diagnosis of respiratory pathogens and is being considered as the new “gold standard”. Although these tests have gained immense popularity, factors such as the patient population (adult, pediatric, and immunocompromised), the size of the laboratory, the purpose of testing (routine or urgent care), and cost/benefit ratio should be considered before implementing a particular assay. For example, during the 2012–2013 influenza outbreak, two nosocomial cases were identified at Memorial Sloan Kettering Cancer Center on January 1, 2013. Despite implementing appropriate infection control measures, five more cases were identified few days later on January 7, 2013. A delay in reporting the positive influenza result was observed as the laboratory was following the routine diagnostic algorithm, and this might have resulted in the subsequent cases of nosocomial infection. Following the 2012–2013 outbreak, the laboratory implemented the FilmArray^®^ RP for routine diagnosis and the Xpert^®^ Xpress Flu/RSV, a rapid molecular POC system, for seasonal use during epidemics and pandemics because the needs for routine testing and urgent care are completely different in these situations. Molecular tests are highly sensitive and specific and result in higher pathogen detection, and therefore physicians and attending clinicians should evaluate test results before initiating a particular course of treatment. Rapid molecular tests are best suited for routine diagnosis and outbreak situations; whereas, conventional methods, such as cell culture or EM could be considered for identifying novel strains of the pathogens.

With the introduction of new technologies, newer assays will be developed for respiratory and other pathogens. For example, using NP swab specimens from children, Graf et al. (2016) demonstrated that untargeted next-generation sequencing-based metagenomics (mNGS) testing had excellent agreement (93%) with multiplex molecular panels and detected more viruses that were either not targeted by the panel or missed due to highly divergent genome sequences. They concluded that mNGS can be used for accurate and unbiased detection of expected or unexpected pathogens. Implementing mNGS downstream of rapid molecular panel testing has been proven useful for characterizing the nature of hospital-associated transmission events and directing the control strategies in pediatric populations during outbreak situations (Greninger et al., 2017). However, diagnostic implementation of NGS is currently limited by incomplete understanding of analytical performance, high cost of the system and complexity of sequence data analysis. Future research will be necessary to focus on demonstrating the clinical utility of these new and upcoming assays in various patient populations and in resource limited settings.

## Author Contributions

All authors listed have made a substantial, direct and intellectual contribution to the work, and approved it for publication.

## Conflict of Interest Statement

SDa and SDu are employees of Luminex Corporation.The remaining author declares that the research was conducted in the absence of any commercial or financial relationships that could be construed as a potential conflict of interest.
